# Management and Treatment Strategies for Distal Tibia and Ankle Infections: Our Clinical Experience

**DOI:** 10.3390/jcm14227967

**Published:** 2025-11-10

**Authors:** Antonio Mascio, Chiara Comisi, Carmen Barlotti, Tommaso Greco, Federico Moretti, Virginia Cinelli, Andrea De Fazio, Giovan Giuseppe Mazzella, Giulio Maccauro, Carlo Perisano

**Affiliations:** 1 Department of Orthopedics and Rheumatological Sciences, Fondazione Policlinico Universitario Agostino Gemelli IRCCS, 00136 Rome, Italy; antonio.mascio87@gmail.com (A.M.); chiara.comisi22@gmail.com (C.C.); federicomoretti2020@gmail.com (F.M.); virginiacinelli23@gmail.com (V.C.); ggmazzella@gmail.com (G.G.M.); carlo.perisano@policlinicogemelli.it (C.P.); 2 Head-Neck and Orthopaedics Sciences, Orthopaedics and Trauma Surgery Unit, Department of Ageing, Neurosciences, Fondazione Policlinico Universitario Agostino Gemelli IRCCS, 00136 Rome, Italy; 3 Department of Life Sciences, Health, and Healthcare Professions, Link Campus University, 00165 Rome, Italy

**Keywords:** osteomyelitis, orthopedic fixation devices, postoperative complications, tibia, ankle, reconstructive surgical procedures, antibiotic therapy

## Abstract

**Background:** The management of infections involving the distal tibia and ankle is a significant challenge in orthopedic surgery due to complex anatomy and the high risk of complications. The study aims to present our clinical experience in managing these infections and focusing on surgical strategies, infection control, and functional outcomes over a minimum 24-month follow-up period. **Methods:** This is an observational, retrospective case series of 17 patients treated for osteoarticular infections of the distal tibia and/or ankle between January 2020 and May 2023, in a second-level referral trauma center. A staged surgical approach was employed, including radical debridement, temporary stabilization with external fixation, and, in most cases, implantation of a cement spacer loaded with antibiotics. Functional outcomes were assessed using scores such as EQ-5D-5L. **Results:** The cohort was predominantly male (76.5%), with a high prevalence of elevated BMI and comorbidities. Infection onset was most frequently associated with open fractures (64.7%). *Staphylococcus aureus* was the most common isolated pathogen (41.2%), and infections caused by Gram-negative or multidrug-resistant bacteria were associated with more reoperations. Overall, complications occurred in 10 patients (58.8%), requiring reintervention in 9 patients (52.9%). Limb salvage was achieved in 16 of 17 patients (94.1%). **Conclusions:** Our study highlights the critical role of a tailored, multidisciplinary approach in managing these complex infections. Meticulous surgical planning and proactive management of complications are essential for optimizing patient outcomes.

## 1. Introduction

Distal tibia and ankle fractures are among the most common injuries in emergency settings due to their complex anatomy and critical role in functional movements [1].

Even more, distal tibial and ankle infections represent a significant challenge in orthopedic surgery and management, particularly in patients with open fractures, impaired soft tissue integrity, or previous unsuccessful treatments [2,3]. The incidence of these infections has increased in recent years, especially in cases involving complex fractures and in patients with multiple comorbidities, such as diabetes and peripheral vascular disease [4,5,6]. Inadequate early management, delayed diagnosis, and insufficient debridement can exacerbate these infections, leading to non-union, chronic osteomyelitis, and potential limb amputation [7,8].

Effective management requires a multidisciplinary approach that integrates accurate skin and cutaneous exposure irrigation and debridement, fracture stabilization, adequate antibiotic therapy, and soft tissue reconstruction [9,10]. The appropriate fixation method option, whether external or internal one, is determined by several factors such as bone loss extension, infection severity, and soft tissue conditions [11,12]. Recent data underscore the benefits of external fixation in managing complex fractures with severe soft tissue damage, although this method is associated with complications like pin site infections and delayed union [10].

Recent studies emphasize the importance of timely surgical intervention, as delayed treatment is often associated with higher infection recurrence and the need for multiple re-interventions [13,14].

Advanced reconstructive techniques, including the Masquelet technique and distraction osteogenesis, as well as custom-made implants and other innovative approaches, have demonstrated good and effective results in managing segmental bone defects in infections [14,15,16].

However, the optimal timing for conversion from external to internal fixation remains debated, with some studies suggesting that early conversion may reduce infection rates, while others recommend prolonged external fixation to ensure infection control [17,18].

Furthermore, antibiotic management is critical in controlling infection, particularly in the context of multidrug-resistant organisms. Empiric broad-spectrum antibiotics are typically initiated, followed by targeted therapy based on microbiological culture results [19]. The emergence of resistant pathogens has underscored the need for tailored antibiotic regimens and adjunctive treatments, such as local antibiotic delivery systems [20].

Therefore, the aim of this study is to present our clinical experience in the management of distal tibia and ankle infections, focusing on surgical strategies, infection control, and functional outcomes over a minimum 24-month follow-up period.

## 2. Materials and Methods

### 2.1. Study Design

This is an observational, retrospective case series about patients treated for distal tibia and/or ankle infections who underwent several surgical treatments at IRCCS Fondazione Policlinico Universitario A. Gemelli (Rome, Italy), a second-level referral trauma center. Data were analysed from September 2024 to May 2025. The recruitment period extended from January 2020 to May 2023, ensuring that all patients included had completed at least 24 months of follow-up by the time of data analysis. At hospital admission, all patients signed a written informant consent concerning demographic and clinical data for scientific purposes according to institutional protocols. The study respects national ethical standards and the Helsinki Declaration.

### 2.2. Inclusion and Exclusion Criteria

Inclusion criteria were (i) patients aged ≥ 18 years; (ii) diagnosis of distal tibial or ankle osteomyelitis, (iii) treated surgically at our institution. Exclusion criteria were (i) previous amputation or non-functional limb; (ii) incomplete data or loss to follow-up.

Inclusion criteria were defined broadly to collect all established osteoarticular infections of the distal tibia and ankle requiring surgical management at our department, including both Fracture-Related Infection (FRI) and primary chronic osteomyelitis without trauma. Patients were categorized according to the FRI Consensus Group definitions [21].

For cases of chronic osteomyelitis unrelated to fractures, diagnostic principles from the Musculoskeletal Infection Society (MSIS) were additionally considered.

### 2.3. Data Collection and Patients Setting

Relevant clinical, demographic, and perioperative data were retrospectively collected from the institutional electronic medical records and operative reports of all patients meeting the inclusion criteria. Data sources included hospital discharge summaries, surgical charts, microbiology and pathology reports, and outpatient follow-up documentation.

Recorded variables included age, sex, Body Mass Index (BMI), smoking status, comorbidities, infection aetiology and localization, microbiological isolate, surgical procedures (both initial and staged), soft tissue reconstruction techniques, complications, and duration of follow-up. Diagnostic imaging data were reviewed to assess bone involvement and infection extension, including X-ray, Computed Tomography (CT) scan, and, when available, Magnetic Resonance Imaging (MRI).

Patients were clinically followed at regular intervals post-operatively. Functional outcome data were collected during in-person evaluations or via telephonic interviews, performed by trained clinical personnel, using the European Quality of Life 5 Dimensions 5 Level Version (EQ-5D-5L). Data consistency was ensured through double entry verification and cross-referencing between medical records and surgical logs. Missing data were minimized and addressed through direct patient contact or review of archived documentation. 

Although the American Orthopaedic Foot and Ankle Society (AOFAS) score, Foot Function Index (FFI), and Short Form-36 (SF-36) questionnaires were initially planned for functional assessment, only the EQ-5D-5L was consistently completed by all patients at final follow-up. Therefore, functional outcome analysis was limited to this score.

All patient information was anonymized prior to statistical processing.

Infection control was defined as the absence of clinical signs or symptoms of infection (such as persistent drainage, erythema, or local pain), normalization of inflammatory markers (C-reactive protein < 10 mg/L and erythrocyte sedimentation rate < 30 mm/h), and radiographic or CT evidence of progressive bone healing or stable consolidation without lytic progression at final follow-up. Patients meeting all three criteria at their last evaluation were considered infection-free.

### 2.4. Outcome Measures

Patients were stratified into two groups based on the time period between the initial injury/osteosynthesis and the definitive staged surgical debridement: acute/subacute infection (treatment initiate days ≤ 90 days post-injury) and chronic post-traumatic osteomyelitis (treatment initiated ≥ 90 days post-injury). The reintervention was defined as any unplanned surgical procedure, excluding minor debridement or pin-site care, performed at the infection site following the definitive first stage of the orthoplastic protocol.

Complications were categorized as either minor or major. Minor complications included local issues that were resolved with conservative or outpatient treatment (e.g., pin tract infections, superficial wound dehiscence). Major complications included recurrence of deep infection, new fractures, failure of reconstruction requiring a completely different treatment strategy, or the need for a major unplanned procedure (e.g., amputation).

### 2.5. Statistical Analysis

Descriptive statistical analysis was conducted using Microsoft Excel 2019 (Microsoft Corporation, Redmond, WA, USA) and Python 3.10.12 (Python Software Foundation, https://www.python.org/, accessed on 16 October 2025) with standard scientific libraries: Pandas 2.1.1, NumPy 1.26.2, Matplotlib 3.8.1, and Seaborn 0.12.2. Continuous variables were expressed as means ± standard deviations (SD) or medians with interquartile ranges (IQR), depending on data distribution. Categorical variables were summarized as absolute frequencies and percentages.

No inferential statistical tests were performed, as the study was designed as a retrospective descriptive case series without a control group. However, to descriptively support specific observations of clinical interest regarding complication risk, focused exploratory comparative analyses (e.g., using Fisher’s exact test for categorical outcomes) were conducted post hoc to investigate the association between the extent of bone resection and complications, as well as surgical duration and complication rates. All other continuous and categorical variables were summarized descriptively, and subgroup comparisons were explored descriptively, particularly regarding reintervention rates, infection aetiology, and surgical procedures.

Functional outcomes based on EQ-5D-5L were analysed by computing mean scores for each domain (mobility, self-care, usual activities, pain/discomfort, and anxiety/depression), and results were reported both individually and in aggregate. Graphical representations, including bar charts, pie charts, histograms, and radar plots, were used to highlight relevant trends in demographics, comorbidity impact on outcomes, microbiology, systemic and local antibiotic regimens, soft tissue reconstruction techniques and success rates, and outcome distribution [22].

## 3. Results

### 3.1. Patient Presentation and Baseline Data

The study cohort consisted of 17 patients diagnosed with chronicsubacute or chronic osteoarticular infections involving the distal tibia and/or ankle, surgically treated between January 2020 and May 2023. All patients met strict inclusion criteria, including a confirmed diagnosis of infection based on clinical, radiological, and microbiological findings, and a minimum follow-up duration of 24 months. 13 patients met the diagnostic criteria for FRI, while four presented with chronic osteomyelitis unrelated to previous fracture or internal fixation. The cohort was predominantly male (13 of 17, 76.5%), reflecting a gender distribution consistent with the higher incidence of post-traumatic and post-surgical musculoskeletal infections in males reported in the literature.The mean age at presentation was 55.3 years (range 32–82; SD ± 13.4), with a bimodal distribution and a peak in the fourth and seventh decades. This reflects the two typical epidemiological profiles most commonly affected: younger patients with high-energy trauma and older patients with multiple comorbidities or previous surgeries.

Anthropometric evaluation showed a BMI of 29.4 (range 18.9–37.5). Eight patients (47%) met the World Health Organization (WHO) criteria for obesity (BMI ≥ 30), and two were morbidly obese (BMI ≥ 35). Given the known association between obesity and increased risk of surgical site complications, impaired vascular supply, and delayed bone healing, the high prevalence of elevated BMI values in this cohort is notable.

Comorbidities were common and clinically relevant. Ten patients (58.8%) had systemic arterial hypertension, while other conditions included chronic obstructive pulmonary disease 4 patients), ischemic heart disease (3 patients), and insulin-dependent diabetes mellitus (3 patients). The clinical significance of these comorbidities was demonstrated by the finding that all diabetic patients developed complications during follow-up, including delayed soft tissue healing and persistent infection, requiring further procedures. Three patients (17.6%) had multiple major comorbidities. Smoking was reported by only two patients, and none were on immunosuppressive therapies or had active malignancies.

Infection occurred following open fracture in 11 patients (64.7%), with fracture patterns corresponding to Gustilo-Anderson grade II or III. Four patients developed infection following previous internal fixation of closed fractures, and two had chronic osteomyelitis without associated trauma. The time between injury and definitive surgical treatment averaged 85 days (range 20–430 days), with three patients presenting after more than one year. Infections treated beyond 90 days from trauma had a significantly higher rate of surgical reintervention (67%) than those treated earlier (37%), showing a strong association with chronicity and treatment complexity (Table 1).

All patients underwent standard imaging protocols, including X-rays and CT scans. MRI was selectively performed in ten patients (58.8%) to evaluate the extension of intramedullary involvement, cortical necrosis, and soft tissue edema.

### 3.2. Treatment Strategies and Outcomes Analysis

All patients were treated with a staged surgical approach based on contemporary principles of infection control and reconstruction. The first stage included radical bone and soft tissue debridement and temporary stabilization using external fixation. Uniplanar Hoffmann III devices were used in less complex cases, while circular or hexapod fixators were applied in patients with unstable fracture patterns, bone loss, or expected length restoration procedures.

The cohort was divided into 10 patients with acute/subacute infection (≤90 days) and 7 patients with chronic post-traumatic osteomyelitis (≥90 days). The reintervention rate was significantly higher in the chronic group: 57.1% (4 of 7) of chronic cases required a subsequent reintervention compared to 37% (3 of 10) in the acute/subacute group (Table 2). The comparison of reintervention rates between the two groups was assessed using Fisher’s Exact Test due to the small sample size; although the chronic osteomyelitis group exhibited a numerically higher reintervention rate (57.1% vs. 30.0%), this difference did not reach statistical significance (*p* = 0.3395).

**Figure 1:**
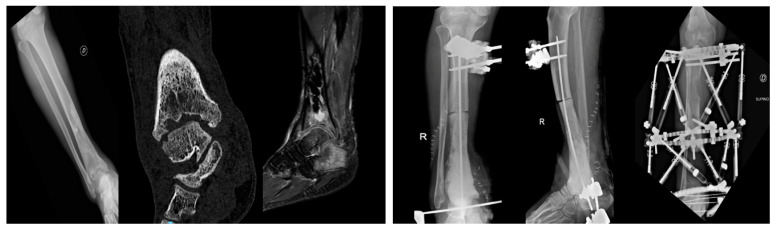

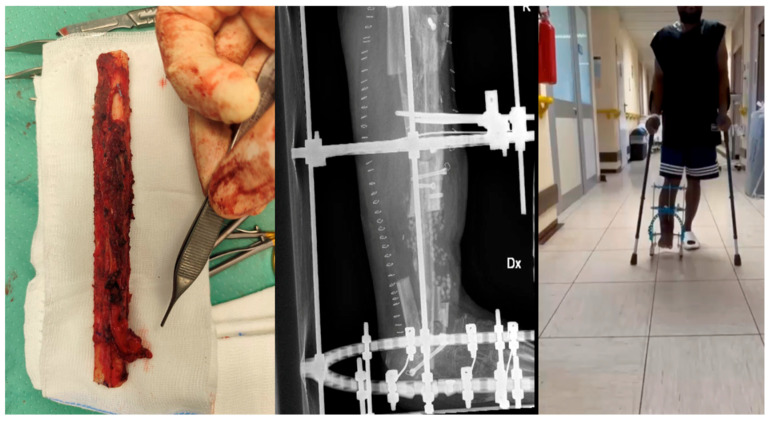
30-year-old male with chronic osteomyelitis of the mid-distal tibia following an undiagnosed and untreated closed trauma for nearly two years. Management included a staged orthoplastic approach: (i) first stage—tibial resection (15 cm) with talar resection, Masquelet technique, and external fixation; (ii) second stage—removal of cement and external fixator with initiation of bone transport; (iii) third stage—fistulectomy, debridement, and reconstruction with a free vascularized fibular flap combined with circular external fixation. Radiographic and clinical signs of healing were achieved, with satisfactory pain control.

In 13 patients (76.4%), a polymethylmethacrylate (PMMA) spacer loaded with antibiotics was implanted. These spacers allowed for temporary defect filling, maintenance of soft tissue tension, and creation of a biologically active membrane consistent with the induced membrane technique, also known as the Masquelet technique. Six patients (35.3%) underwent bone resection exceeding 5 cm, with one patient requiring removal of a 15 cm segment of the tibial diaphysis. In all these cases, the Masquelet technique was applied to promote membrane formation prior to delayed reconstruction. One patient with massive segmental loss underwent reconstruction using a vascularized fibular graft in combination with circular external fixation, demonstrating the utility of advanced orthoplastic techniques in high-demand cases (Figure 1).

Antibiotics were mixed with cement spacers based on microbiological data and included vancomycin, gentamicin, and tobramycin. In five cases, local therapy was enhanced by bioabsorbable carriers such as calcium sulphate beads.

Soft tissue reconstruction was required in 15 patients (88.2%). Sural fasciocutaneous flaps were used in 9 cases, while 3 patients underwent perforator-based flap reconstruction. Two patients received musculocutaneous gastrocnemius flaps, and 1 patient received a vascularized fibular bone and soft tissue flap, reflecting the need for individualized treatment strategies in extensive defects. Overall, the majority of soft tissue reconstruction attempts were successful, with flap-related complications occurring in only 3 patients (20% of reconstructed cases), including minor necrosis and delayed healing, all of which were managed successfully without requiring a complete flap failure reintervention.

Microbiological cultures were positive in all cases. The most frequently isolated organism was *Staphylococcus aureus* (seven patients, 41.2%), including MRSA and MSSA strains. *Pseudomonas aeruginosa* was cultured in 3 patients, each requiring at least one reintervention. Other organisms included *Cutibacterium acnes*, *Acinetobacter pittii*, *Enterococcus faecalis*, *Klebsiella pneumoniae*, and *Corynebacterium striatum*. Polymicrobial infections occurred in 7 cases. One patient had a rare pathogen, *Wohlfahrtiimonas chitiniclastica*, complicating diagnosis and therapy. Infections caused by Gram-negative or multidrug-resistant bacteria were associated with longer treatment durations and more reoperations.

Systemic antibiotic regimens were selected in collaboration with infectious disease specialists and were tailored to the organism identified. A key component of our protocol was the use of Daptomycin, which was the most frequently used agent (11 patients), often in combination with rifampicin, cefepime, or meropenem. Overall, the majority of the cohort (12 patients, 70.6%) received combined systemic and local antimicrobial treatment, with therapy continued for more than 12 weeks in 10 patients (58.8%). This combined approach underscores the aggressive nature of treatment for these resistant pathogens.

Complications were reported in 10 patients (58.8%), the most common being persistent draining fistulas (5 patients), non-union (4 patients), and implant failure (1patient). Reintervention was necessary in 9 patients (52.9%), primarily among those with infections due to Pseudomonas aeruginosa or MRSA, and among those undergoing arthrodesis or bone transport. Patients with polymicrobial or resistant infections had the highest risk of failure. Limb salvage was achieved in 16 patients (94.1%). Therefore, minor complications accounted for 47.1% (8 of 17) of the cohort and including 6 cases of pin tract infection and 2 cases of superficial wound dehiscence, all managed conservatively. Major complications accounted for 11.7% (2 of 17) of the cohort, comprising one case of deep re-infection requiring repeat debridement and the single case resulting in below-knee amputation.

Second-stage surgical procedures were performed in 10 patients. Autologous iliac crest bone grafts were used in four patients, while two received cadaveric grafts. The remaining cases used synthetic or composite substitutes. Seven patients underwent tibiotalar or tibiocalcaneal arthrodesis using either retrograde nails or plates. Two patients underwent bone transport with corticotomy and distraction through circular frames. One patient ultimately required transtibial amputation due to combined failure of soft tissue coverage, infection control, and fixation (Table 3).

Functional recovery and health-related quality of life were assessed at final follow-up using the self-reported EQ-5D-5L questionnaire. It is a standardized, non-disease-specific Patient-Reported Outcome Measures (PROMs) used internationally to assess an individual’s Health-Related Quality of Life (HRQoL). It is frequently employed in clinical trials, economic evaluations, and health policy decisions due to its simplicity and robust psychometric properties [23]. The most impaired domains were “usual activities” (mean 3.4), “mobility” (3.1), and “pain/discomfort” (2.9). “Self-care” and “anxiety/depression” were less severely impacted. Patients who required multiple surgeries, especially for infection recurrence, reported lower functional scores overall. These findings are consistent with published data on the long-term morbidity of chronic post-traumatic osteomyelitis (Table 4).

## 4. Discussion

The management of infections involving the distal tibia and ankle remains a significant challenge in orthopedic surgery. These complex cases require a multidisciplinary approach that integrates orthopedic and plastic surgery principles to optimize outcomes. Key components of successful treatment include meticulous debridement, stable skeletal fixation, and effective soft tissue coverage [2,3].

**Figure 2:**
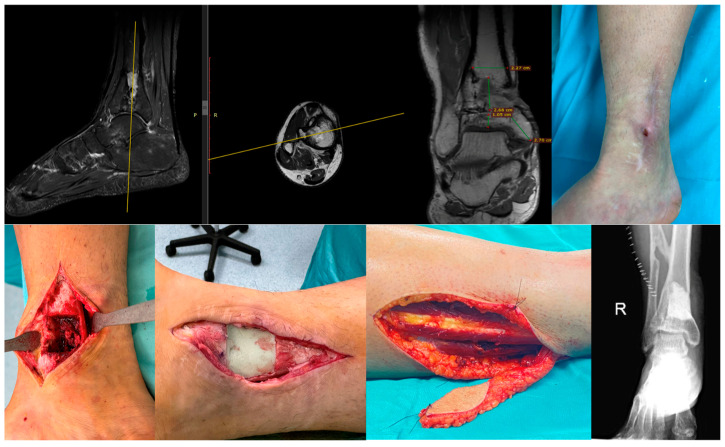
39-year-old male with a history of distal tibial metaphyseal fracture treated with ORIF, complicated by chronic osteomyelitis. He underwent one single stage surgical procedure including fistulectomy, partial tibial resection with 3 × 5 cm cortical window, debridement, and antibiotic cement placement, covered with a peroneal perforator flap.

Within our cohort, several highly complex cases were managed through coordinated collaboration between orthopedic surgeons, infectious disease specialists, plastic surgeons, and interventional radiologists (Figure 2). Our findings underscore the critical impact of chronicity on the treatment complexity, demonstrating that infections treated beyond 90 days from the initial trauma were associated with a notably higher surgical reintervention rate (67% vs. 37%). This observation aligns closely with established literature. Similarly, Niebuhr et al., in their systematic review, identified chronic infection as a major risk factor for subsequent treatment failure [24].

In our cohort, external fixation was as the most frequently employed surgical technique, particularly in cases characterized by severe bone loss and multi-fragmentary fractures. This approach is supported by previous studies demonstrating the efficacy of external fixation in managing of complex distal tibial fractures while minimizing disruption of soft tissues [25]. However, our findings showed higher incidence of complications associated with external fixation, notably fistulas and hardware exposure, in line with complication rates reported in the literature ranging from 7% to 32% [7].

Our overall complication rate (58.8%) aligns with the study of Brauns et al., who observed a 55% reoperation rate in infected pilon non-unions [26]. However, our limb salvage rate (94.1%) was higher than the 85–90% typically reported in similar series, possibly reflecting early multidisciplinary management [27,28]. This case involved a patient presenting with an extremely chronic, polymicrobial infection (including resistant *Pseudomonas*), coupled with severe, uncontrolled diabetes mellitus and extensive vascular compromise precluding successful revascularization and reliable free flap coverage. Despite aggressive debridement and multiple attempts at infection control, the combination of a persistently non-healing soft tissue envelope, progressive bony necrosis, and significant patient-reported pain led to the final decision for below-knee amputation, prioritizing patient function and definitive infection clearance.

A recent study by Sambri et al. evaluated 13 patients with infection-related distal tibial fractures managed through an orthoplastic approach, including antibiotic-loaded spacers and vascularized flaps. At a follow-up of 25 months, no recurrences of infection were observed, suggesting the efficacy of a comprehensive orthoplastic protocol in infection control [27]. Compared with this study, our results confirm that combined orthoplastic management achieves infection control rates exceeding 90%, despite variable reconstructive techniques.

Conversely, the utilization of plates and bone transport techniques demonstrated lower complication rates. The strategic application of these methods in less complex fracture patterns or as part of a staged reconstruction protocol may account for the favourable outcomes observed [8]. A case report involving the use of the Masquelet technique combined with a PRECICE nail for segmental bone defects further underscores the utility of staged reconstructive procedures in managing post-traumatic infections [4].

Our exploratory analysis revealed a statistically significant correlation between the extent of bone resection and the occurrence of complications (*p* = 0.0043), highlighting a strong association within this cohort. Larger resections pose challenges in achieving stable fixation and adequate soft tissue coverage, thereby increasing the risk of postoperative complications [24]. Additionally, prolonged orthoplastic procedures were significantly associated with higher complication rates (*p* = 0.0017), suggesting that extended surgical time reflects the level of procedural complexity and potential morbidity in these challenging cases [25].

A study on the implementation of an orthoplastic treatment protocol for open tibial fractures demonstrated a marked reduction in infection rates from 20.6% to 1.6%, further reinforcing the importance of a structured, multidisciplinary approach in managing complex tibial infections [28].

The demographic profile of our patient population, characterized by a mean age of 47.2 years and a male predominance (68%), reflects the epidemiological trends observed in distal tibial and ankle infections [29]. While our study did not find a statistically significant association between sex and fracture frequency (*p* = 0.3856), the higher prevalence in males warrants further investigation into potential occupational or behavioural risk factors.

The retrospective nature of our study and the relatively small sample size limit the generalizability of our findings. Moreover, given the retrospective and descriptive nature of our study, all associations should be interpreted as correlations rather than causal relationships. In fact, while our analysis identified strong associations between factors such as the timing of surgery (beyond 90 days), the extent of bone resection, and the presence of complications, these findings represent correlations within our cohort and do not establish a causal relationship. Future prospective studies are necessary to validate these observations and confirm the prognostic value of these factors.

One other limitation of this study is that incomplete data for AOFAS, FFI, and SF-36 scores prevented their inclusion in the analysis. Moreover, the integration of advanced imaging modalities and biomarker analyses may enhance the early detection of complications and inform personalized treatment strategies.

## 5. Conclusions

Our study underscores the critical role of a tailored, multidisciplinary approach in managing distal tibial and ankle infections. The selection of surgical techniques should be guided by the complexity of the fracture, the extent of bone and soft tissue involvement, and patient-specific factors. External fixation was the most frequently employed technique in this cohort, primarily due to the complexity of the cases rather than superior efficacy. Given the observed rate of complications, its role should be considered within a staged, multidisciplinary treatment algorithm rather than as a standalone solution. Careful patient selection, appropriate timing of conversion to internal fixation, and coordinated orthoplastic management remain key to optimizing outcomes and minimizing recurrent infection.

## Figures and Tables

**Table 1:** Demographic and clinical characteristics of the study cohort, including age, sex distribution, BMI, comorbidities, etiology of infection, and baseline clinical status. These variables provide an overview of the patient population treated for distal tibia and ankle infections in our institution.

Gender	*n* (%)
Male	13 (76.5)
Female	4 (23.5)
**Age**	**Years**
Mean	55.3
Range	32–82; SD ± 13.4
**BMI**	**Value**
Mean	29.4
Range	18.9–37.5
Obese (BMI ≥ 30)	8 (47%)
Morbidly obese (BMI ≥ 35)	2
**Comorbidity**	***n* (%)**
Hypertension	10 (58.8)
COPD	4 (23.5)
Ischemic heart disease	3 (17.6)
Diabetes mellitus (insulin-dependent)	3 (17.6)
Multiple comorbidities	3 (17.6)
Smokers	2 (11.8)
**Aetiology**	***n* (%)**
Open fracture (Gustilo II/III)	11 (64.7)
Post-internal fixation of closed fracture	4 (23.5)
Chronic osteomyelitis (no trauma)	2 (11.8)

**Table 2:** Comparison of the rate of reintervation betweens patients treated early (≤90 days) and those with chronic post-traumatic osteomyelitis (≥90 days). The comparison of reintervention rates between the two groups was assessed using Fisher’S Exact Test due to the small sample size (*p* = 0.3395).

Infection Category	Patients (*n* = 17)	Reintervention (Yes)	Reintervention (No)	Rate of Reintervention
**Acute/Subacute** (≤90 days)	10	3	7	**30.0%**
**Chronic** (≥90 days)	7	4	3	**57.1%**
**Total**	**17**	**7**	**10**	

**Table 3:** Laboratory and type of treatment of the study cohort, including isolated pathogen microorganism, antibiotic therapy, bone and soft tissues reconstruction techniques, following by all complications registered in almost 24 months of follow-up.

Isolated Pathogens	*n* (%)
Staphylococcus aureus (MRSA/MSSA)	7 (41.2)
Pseudomonas aeruginosa	3 (17.6)
Cutibacterium acnes	1 (5.9)
Acinetobacter pittii	1 (5.9)
Enterococcus faecalis	1 (5.9)
Klebsiella pneumoniae	1 (5.9)
Corynebacterium striatum	1 (5.9)
Wohlfahrtiimonas chitiniclastica	1 (5.9)
Polymicrobial infections	7 (41.2)
**Antibiotic Regimen**	***n* (%)**
Systemic only	5 (29.4)
Systemic + local	12 (70.6)
Therapy > 12 weeks	10 (58.8)
**Soft Tissue Reconstruction Techniques**	***n* (%)**
Sural fasciocutaneous flap	9 (52.9)
Perforator-based flap	3 (17.6)
Gastrocnemius musculocutaneous flap	2 (11.8)
Vascularized fibular bone + soft tissue flap	1 (5.9)
**Bone Reconstruction Techniques**	***n* (%)**
Autologous iliac crest graft	4 (23.5)
Cadaveric graft	2 (11.8)
Arthrodesis (tibiotalar/tibiocalcaneal)	7 (41.2)
Bone transport	2 (11.8)
Amputation	1 (5.9)
**Complications**	***n* (%)**
Persistent draining fistulas	5 (29.4)
Non-union	4 (23.5)
Implant failure	1 (5.9)
Any complication (total)	10 (58.8)
Reintervention required	9 (52.9)

**Table 4:** Functional outcomes were evaluated using the EQ-5D-5L index at final follow-up.

EQ-5D-5L Domains	Mean Score
Mobility	3.1
Self-care	2.1
Usual activities	3.4
Pain/Discomfort	2.9
Anxiety/Depression	2.3

## Data Availability

The original contributions presented in this study are included in the article. Further inquiries can be directed to the corresponding author.

## Abbreviations

The following abbreviations are used in this manuscript:

AOFAS	Orthopaedic Foot & Ankle Society
BMI	Body Mass Index
CT	Computed Tomography
EQ-5D-5L	European Quality of Life 5 Dimensions 5 Level Version
FFI	Foot Function Index
FRI	Fracture-Related Infection
IQR	Interquartile Range
MRI	Magnetic Resonance Imaging
MRSA	methicillin-resistant *Staphylococcus aureus*
MSSA	methicillin-susceptible *Staphylococcus aureus*
PMMA	Polymethylmethacrylate
SD	Standard Deviation
SF	Short Form
WHO	World Health Organization
